# Consistency of the Tools That Predict the Impact of Single Nucleotide Variants (SNVs) on Gene Functionality: The *BRCA1* Gene

**DOI:** 10.3390/biom10030475

**Published:** 2020-03-20

**Authors:** Javier Murillo, Flavio Spetale, Serge Guillaume, Pilar Bulacio, Ignacio Garcia Labari, Olivier Cailloux, Sebastien Destercke, Elizabeth Tapia

**Affiliations:** 1 Centro Internacional Franco Argentino de Ciencias de la Información y de Sistemas (CIFASIS-CONICET), Universidad Nacional de Rosario, CP 2000 Rosario, Santa Fe, Argentina; spetale@cifasis-conicet.gov.ar (F.S.); bulacio@cifasis-conicet.gov.ar (P.B.); ignaciogarcialabari@gmail.com (I.G.L.); tapia@cifasis-conicet.gov.ar (E.T.); 2 ITAP, Univ Montpellier, INRAE, Montpellier SupAgro, Montpellier, France; serge.guillaume@irstea.fr; 3 Université Paris-Dauphine, Université PSL, CNRS, LAMSADE, 75016 Paris, France; olivier.cailloux@dauphine.fr; 4 Université de Technologie de Compiegne, 60200 Compiegne, France; sebastien.destercke@hds.utc.fr

**Keywords:** SNV, prediction tools, *BRCA1* gene, consistency of tools, preference relations

## Abstract

Single nucleotide variants (SNVs) occurring in a protein coding gene may disrupt its function in multiple ways. Predicting this disruption has been recognized as an important problem in bioinformatics research. Many tools, hereafter p-tools, have been designed to perform these predictions and many of them are now of common use in scientific research, even in clinical applications. This highlights the importance of understanding the semantics of their outputs. To shed light on this issue, two questions are formulated, (i) do p-tools provide similar predictions? (inner consistency), and (ii) are these predictions consistent with the literature? (outer consistency). To answer these, six p-tools are evaluated with exhaustive SNV datasets from the *BRCA1* gene. Two indices, called $K_{all}$ and $K_{strong}$, are proposed to quantify the inner consistency of pairs of p-tools while the outer consistency is quantified by standard information retrieval metrics. While the inner consistency analysis reveals that most of the p-tools are not consistent with each other, the outer consistency analysis reveals they are characterized by a low prediction performance. Although this result highlights the need of improving the prediction performance of individual p-tools, the inner consistency results pave the way to the systematic design of truly diverse ensembles of p-tools that can overcome the limitations of individual members.

## 1. Introduction

To fulfill its biological function under specific environmental conditions, such as the cellular milieu, each protein must be folded into a defined three-dimensional structure, known as its native structure. Structural modifications of proteins may result in partial or total loss of function, as in the case of cystic fibrosis disease [1,2]. These modifications can also be harmful to the cell for reasons not directly related to protein function, as in the case of Alzheimer’s, Parkinson’s, and Huntington’s disease [3,4], where misfolded proteins bind together into aggregates that accumulate and are toxic for the cell. One of the main factors underlying the conformation of a protein is the amino acid sequence. A change in an individual nucleotide (also known as a single nucleotide variant or SNV) in a protein coding gene may lead to an amino acid change. In this case, the SNV involves a non-synonymous substitution, called a missense mutation. In other cases, a SNV may produce a premature stop codon leading to protein truncation, in what is known as a nonsense mutation.

Predicting the degree to which an SNV impacts protein function is an important and challenging problem in bioinformatics research. The development of next generation sequencing technologies has made it possible to detect thousands of missense SNVs in protein-coding genes [5]. Although the wet-lab testing of these SNVs to determine their functional and physiological effects remains unaffordable, it has been recognized [6] that the ability to discriminate between harmful and benign mutations in silico could significantly reduce the set of SNVs warranting deeper studies.

Thus far, many bioinformatics tools, hereafter referred to as p-tools, have been developed to predict the effect of SNVs in protein function as reviewed in [7]. In this regard, sequence conservation analysis is one of the most commonly used strategies. Multiple sequence alignments allow the identification of amino acids conserved through evolution. These amino acids are likely to be important for protein function, so substitutions on these positions are expected to have severe impacts on protein function, especially if the substitutions involve amino acids with different physico-chemical properties. In addition, structural information is also used to infer the sites where amino acid substitutions are more likely to have a negative impact on the protein function. Similarly, changes in amino acids characterizing secondary structures are expected to severely impact protein function. It follows from the foregoing that the degree to which SNVs impact protein function depends on the specific amino acid change, its relative position within the protein, and the protein context.

We note, however, that the way p-tools perform and communicate predictions is highly variable, even for those relying on conceptually similar third-party methods. So, even if p-tools prove to be useful, which one should we use? One of the main problems that researchers bump into when trying to compare p-tools is the interpretation of their outputs. Let us consider three well known instances: Panther [8], Strum [9], and Polyphen2 [10]. Panther provides only a categorical output indicating the possibility of a SNV being damaging, Strum provides only a numerical value indicating the change in protein fold stability, and Polyphen2 provides a categorical output together with a probability value.

The variety of outputs make the task of comparing p-tools (i.e., for determining inner consistency) tricky. To overcome this problem, a straightforward approach would be to transform all p-tool outputs to a predefined common set of categories. For p-tools already including categorical outputs, this means defining a convenient mapping between set of categories. On the other hand, for p-tools involving just numerical outputs, convenient thresholds are required. This is the approach taken in [11], where SNVs predictions from different p-tools are reclassified into three categories, namely, unknown effect, neutral and possibly pathogenic, with the aim of evaluating their inner consistency. However, these transformation-based approaches may not only reduce the information content of p-tool outputs but may bias inner consistency results due to mapping and threshold dependencies.

Ideally, comparisons between p-tools should be performed without intermediary transformations, despite differences in the output scales and in the ranges of their native categories. In this line of research, we introduce two novel indices, called $K_{all}$ and $K_{strong}$, to assess the inner consistency of p-tools. None of these requires the transformation of the p-tool outputs. They only require that the p-tools assess the relative impact of SNV pairs, i.e., is mutation *m* less damaging than $m^{\prime }$? The fraction of SNV pairs receiving contradictory orderings is used to quantify the degree of disagreement between pairs of p-tools and thus, their inner consistency. Briefly, the $K_{all}$ index counts all type of disagreements between p-tools while the $K_{strong}$ index only counts disagreements involving opposite predictions. As a result, the $K_{all}$ and $K_{strong}$ indices enable the systematic and intuitive comparison of p-tools. Beyond inner consistency, it is desirable for predictions by p-tools to match the existing literature results, i.e., to have good outer consistency. To assess outer consistency, p-tools were evaluated with standard information retrieval metrics [12,13] including accuracy, precision, recall, the F1-score, and the Matthews correlation coefficient (MCC).

Six p-tools using different information sources and prediction logic were selected based on their popularity in the scientific community and the possibility of being executed online. The inner and outer consistency of the selected p-tools were evaluated against two particular datasets involving the breast cancer type one susceptibility protein encoded by the *BRCA1* gene. Both datasets comprise in vitro experiments allowing the exhaustive screening of *BRCA1* mutation effects [14]. The first dataset comprises roughly 4000 SNVs on 1792 nucleotide positions generated by means of the saturation genome editing (SGE) technique [15] relying on the CRISPR-Cas9 technology.

The second dataset comprises 1056 amino acid mutations in the first 191 residues of the BRCA1 protein generated by means of site-saturation mutagenesis were the authors [16] perform a multiplex homology-directed DNA repair assay designed to test whether homology-directed repair (HDR) [17] of double-strand DNA breaks occurs in *BRCA1* mutant cells. Due to its CRISPR-Cas9 foundation, the SGE technique may induce multiple genetic mutations beyond the desired one. These undesired mutations may compromise the viability of cells beyond the effect of the SNVs under study. As a result, conclusions concerning the pathogenecity of *BRCA1* SNVs drawn from SGE could, in principle, be biased. Fortunately, this does not appear to be the case and the results reported in [15] are in good agreement with those reported in [16], confirming the value of the SGE technique for performing high throughput studies into the effect of SNVs.

From a computational point of view, the SGE technique provides exhaustive and unbiased SNV datasets as every gene position can be tested for all possible mutations. In addition, site-saturation mutagenesis allows the generation of exhaustive and unbiased single-amino acid mutagenesis datasets for the BRCA1 protein. Although only a fraction of these mutations are accessible by SNVs relevant to human disease, the information content of the whole dataset is definitively higher and thus better for evaluation studies of p-tools. On the whole, the availability of exhaustive and unbiased datasets of SNVs or mutated amino acids remarkably simplifies and normalizes the evaluation of p-tools. To the best of our knowledge, the public availability of SGE datasets is currently limited to the *BRCA1* gene. This gene belongs to the ‘first wave’ of susceptibility genes for common types of cancer [18]. Therefore, the identification of carriers of pathogenic mutations in this gene is expected to be more impactful for cancer control.

## 2. Materials and Methods

### 2.1. P-Tools

The effect of SNVs on the functionality of the *BRCA1* gene was assessed by means of the PolyPhen2 [10], the Provean [19], the Align GVGD [20], the Strum [9], the Cupsat [21], and the Panther [8] prediction tools. In all cases, except for PolyPhen2, in which we used the HumVar classification model (advanced options), which was better suited for this study, their online version configured with default parameters were used. For Cupsat predictions, the Protein Data Bank (PDB) file of BRCA1 was provided. Further details about the selected p-tools can be found in the Appendix A.

### 2.2. Datasets

*BRCA1-SGE* dataset. The authors [15] studied the ability to grow haploid human cells in cell cultures. Cells were edited by means of the CRISPR-Cas9 technology with a focus on every nucleotide (saturation genome editing) of the *BRCA1* gene in a region spanning 13 different exons known to encode critical functional domains. The original study comprises nearly 4000 mutations belonging to exons 2–5 and 15–23, including some adjacent intron sequence. Cultured cells that managed to survive to gene editing were considered to hold a functional BRCA1 protein. The original dataset was filtered to remove misleading SNVs classified as “Likely Benign” missense mutations. As a result, the final dataset comprises 387 “pathogenic” missense SNVs (positive examples) and 1405 “benign” missense ones (negative examples).

*BRCA1-HDR* dataset. The authors [16] performed a Multiplex Homology-Directed Repair Assay with the aim of quantifying the effect of 1056 amino acid substitutions in the BRCA1 N terminus comprising residues 2–192 known to include the ring domain in residues 7–98. As proper folding of the RING domain is required for the stability and function of the full-length protein, the authors analyze whether the mutated BRCA1 protein is able to maintain its DNA repair function in the homology-directed repair (HDR) pathway using, in tissue culture, a green fluorescent protein (GFP) based reporter assay [17] in which the functionality of BRCA1 can be detected by identifying green-flourescent cells. The information about the impact of amino acid mutations on the HDR pathway was depicted graphically using a color scale.

An in house R [22] script was used to convert the graphical information to a plain text format. Based on the depletion scores (fluorescence drops respect to a subset of cells having a functional GFP allele encoding an active protein) observed across four replicates of the multiplex HDR reporter assay, mutations showing a depletion in none or just one replicate were considered “benign” (negative examples). On the other hand, mutations showing a depletion state in at least three replicates were considered “pathogenic”; mutations showing depletion states involving two replicates were discarded. As a result, the final dataset comprises 59 “Pathogenic” variants (positive examples) and 977 “benign” ones (negative examples).

As expected, both datasets turned out to be highly imbalanced with most of the mutations being of the “benign” type. To quantify the degree of data imbalance, the relative gap $G=\frac{\#pathogenic-\#benign}{\#mutations}$ between positive and negative examples was computed for each dataset. *G* values of −0.56 and −0.88 were observed for the *BRCA1-SGE* and *BRCA1-HDR* datasets, respectively.

### 2.3. Inner Consistency Analysis

The task of assessing the inner consistency of the p-tools faces the problem of the heterogeneity of their outputs. It is not simply a problem of outputs involving different scales but of their semantic meaning. Usually, p-tools provide categories to classify the impact of mutations on the functionality of a gene. However, these categories are not equally distributed through their original numerical scales, thus conversions made by the tools are not linear. Furthermore, different numerical scales are used, from probabilities and free-energy values, to ad-hoc scores. Hence, normalization approaches do not make sense. We note, however, that once categories are defined for a p-tool, they naturally induce an internal ranking for numerical predictions. Given a pair of p-tools and a dataset of mutations, the agreement between their internal rankings can be used to assess their inner consistency.

Under this baseline, we first considered the Kendall rank correlation coefficient (τ) [23] measuring the ordinal association between two measured quantities. Briefly, given a pair of p-tools and a set of target mutations, high values of τ are expected whenever target mutations receive similar ranks in both tools. Formally, let $M=\{m_{1},m_{2},\ldots ,m_{i},\ldots ,m_{j},\ldots ,m_{n}\}$ be a set of mutations with *n* being the number of mutation sites multiplied by the number of allowed mutations per site. Also, let $t_{S}\left(m\right):M\to X_{S}$ denote the effect of mutation *m* predicted by a given p-tool *S* with $X_{S}$ be the most informative scale provided by *S*. In addition, let ${≺}_{S}\subseteq M\times M$ be the *less-damaging-than relation* induced by *S* on mutations $m_{i}$ and $m_{j}$ so that $m_{i}{≺}_{S}m_{j}$ if $t_{S}\left(m_{i}\right)<t_{S}\left(m_{j}\right)$, i<j≤n. Finally, to simplify the notation, for any p-tool *S*, three orderings are possible for any pair of mutations $m_{i}$ and $m_{j}$, namely, $m_{i}≺m_{j}$, $m_{i}≻m_{j}$, and $m_{i}∼m_{j}$, i<j≤n.

A concordant pair of predictions for p-tools *S* and *P* is accounted whenever $m_{i}≻m_{j}$ or $m_{i}≺m_{j}$ occurs for both *S* and *P*, i<j≤n. Conversely, a discordant pair of predictions is accounted for p-tools *S* and *P* whenever $m_{i}≻m_{j}$ occurs for P(S) and $m_{i}≺m_{j}$ occurs for S(P), i<j≤n. Alternatively, if $m_{i}∼m_{j}$ occurs for either *S* or *P*, a neither concordant nor discordant pair of predictions is accounted, i<j≤n. Based on these considerations, the Kendall τ coefficient can be defined as follows:

τ=1−(# concordant pairs)−(# discordant pairs)n2

P-tools with native numerical outputs provide convenient categorical outputs by the adoption of sharp thresholds. This common practice may induce false concordant/discordant pairs in the Kendall τ computation which misleads the comparison of p-tools. For example, let us consider [0, 0.4] being the support of the category label “Benign” with predictions in the [0, 1] range. Intuitively, prediction values of 0.39 and 0.41 are so close that we may not use them to differentiate categories of mutation effects. Hence, although the Kendall τ coefficient can be used with p-tools numerical outputs, its value for measuring the inner consistency of p-tools raises some concerns.

Furthermore, the numerical outputs of p-tools may differ due to computational precision issues, additionally inducing false concordant/discordant pairs in the Kendall τ computation that further misleads the quantification of the inner consistency of p-tools. In brief, the Kendall τ coefficient appears too “sensitive” to assess the inner consistency of p-tools with numerical outputs. To overcome this problem, let us first define a convenient function $r_{S}\left(m_{i},m_{j}\right)$ characterizing the specific ordering assigned to mutations $m_{i}$ and $m_{j}$, i<j≤n, by any p-tool *S*:

(1) $$r_{S}\left(m_{i},m_{j}\right)=\left\{\begin{array}{cc} 1 & \mathrm{if}\;m_{i}{≺}_{S}m_{j}\\ -1 & \mathrm{if}\;m_{i}{≻}_{S}m_{j}\\ 0 & \mathrm{if}\;m_{i}{∼}_{S}m_{j} \end{array}.\right.$$

We now introduce a novel index, called $K_{all}$, able to properly account for all different prediction pairs issued by p-tools *S* and *P*:

(2) Kall=1−|(mi,mj)rS(mi,mj)≠rP(mi,mj)|n2.

For p-tools involving native categorical outputs, category labels are ordered based on their impact on gene functionality, e.g., for category labels {benign,possibly,probably}, the preference relation benign≺possibly≺probably is assumed. On the other hand, for p-tools involving numerical outputs, equality δ>0 thresholds are required to avoid the false counting of either concordant or discordant pairs. Let *S* be a p-tool with a numerical output and an equality threshold $\delta_{S}$. Hence, the preference of *S* on mutations $m_{i}$ and $m_{j}$, i<j≤n, is defined as follows:

$$m_{i}{≺}_{S}m_{j}\Leftrightarrow t_{S}\left(m_{j}\right)-t_{S}\left(m_{i}\right)>\delta_{S},\;\delta_{S}\geq 0.$$

Hence, $m_{i}{∼}_{S}m_{j}\Leftrightarrow \left|t_{s}\left(m_{j}\right)-t_{s}\left(m_{i}\right)\right|\leq \delta_{S}$. Since p-tools generally involve different prediction ranges, their thresholds must be set accordingly. In the absence of prior information, setting these thresholds to some predefined percentage of their prediction ranges appears as a fair approach. The problem becomes how to set that percentage. At first glance, the thresholds must be large enough to avoid small prediction differences and numerical errors to induce discordant counts, but also small enough to avoid the false counting of either concordant or discordant pairs.

To shed light on the percentage equality threshold trade-off problem, let us consider the mutations $m_{i}$ and $m_{j}$, i<j≤n, and the predictions issued by the tools *S* and *P*. Let us consider first the case where $m_{i}≺m_{j}$ holds for both tools. Also, let us define $\Delta_{S}=\left|t_{S}\left(m_{i}\right)-t_{S}\left(m_{j}\right)\right|$ and $\Delta_{P}=\left|t_{P}\left(m_{i}\right)-t_{P}\left(m_{j}\right)\right|$, $\Delta_{S}$ < $\Delta_{P}$. If $\delta <\Delta_{S}$, then $m_{i}{≺}_{S}m_{j}$ and $m_{i}{≺}_{P}m_{j}$ so that an agreement is counted for $K_{all}$. However, if $\Delta_{S}\leq \delta <\Delta_{P}$, then $m_{i}{∼}_{S}m_{j}$ and $m_{i}{≺}_{P}m_{j}$, so that a disagreement is counted for $K_{all}$. However, if $\delta \geq \Delta_{P}$, then $m_{i}{∼}_{S}m_{j}$ and $m_{i}{∼}_{P}m_{j}$, so that an agreement is counted for $K_{all}$ again.

Similar counting arguments can be used to analyze the cases $m_{j}{≺}_{S}m_{i}$ and $m_{i}{≺}_{P}m_{j}$. In all cases, as the percentage equality threshold is increased from 0%. $K_{all}$ first decreases and then increases monotonically until the percentage equality threshold reaches 100%. All mutations then become indistinguishable and $K_{all}$ reaches its maximum value (1). To summarize, $K_{all}$ does not show a monotonic behavior with respect to the percentage equality threshold. Supplementary studies were performed to asses the critical percentage equality threshold where $K_{all}$ accomplishes its minimum.

**Figure 1:**
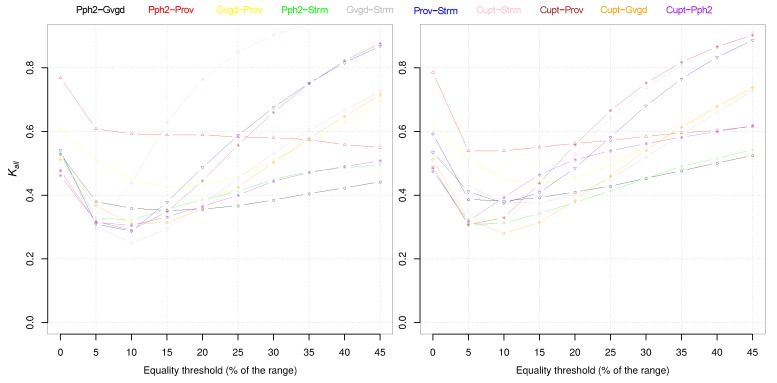
Threshold analysis. (**left**) *DM-V* dataset and (**right**) *CHKV-E2* dataset.

Two independent datasets of mutations, namely, the *DM-V* dataset comprising reported mutations of the *Drosophila melanogaster vermilion (V)* gene and the *CHKV-E2* dataset comprising reported mutations of the *Chikongunya* virus *E2* gene, were used to evaluate the $K_{all}$ index with respect to increasing values of the percentage equality threshold. All p-tools were analyzed except Panther as this only provides a categorical output. As a result (see Figure 1), the percentage equality threshold was set to 5%, with an intermediate value between 0% (no threshold) and that value where (∼10%) $K_{all}$ falls to its minimum.

Users of p-tools might be additionally interested in the identification of pairs of p-tools showing not only a considerable proportion of disagreements but a particular form of them, that involving opposite predictions, i.e., $m_{i}{≺}_{S}m_{j}$ and $m_{j}{≺}_{P}m_{i}$. In this case, the $K_{strong}$ index can be used:

(3) Kstrong=1−|(mi,mj)rS(mi,mj)≠0∧rS(mi,mj)=−rP(mi,mj)|n2.

While the $K_{all}$ index measures the proportion of pairs of predictions for which conflicting orderings are observed, the $K_{strong}$ index focuses only on extreme conflicting orderings. In practice, users might use the $K_{all}$ index for the identification of similar p-tools looking for $K_{all}$ values close to one. Conversely, users might use the $K_{strong}$ index for the identification of different p-tools looking for $K_{strong}$ values close to zero. Beyond these considerations, the ranges and the directions of $K_{strong}$ and $K_{all}$ are similar so that values closed to 1 indicate that pair of p-tools are likely to order all pairs of mutations in a similar way, while values closed to 0 indicate they are likely to order them differently. Similar counting arguments to those used with the $K_{all}$ index, can be used to asses the effect of percentage equality thresholds on the $K_{strong}$ index. Differently from $K_{all}$, a monotonic decreasing behaviour is observed for $K_{strong}$ for increasing values of the percentage equality threshold. However, since we expect that $K_{strong}$ only dissects the inner consistency information already provided by its more general $K_{all}$ counterpart, practical $K_{strong}$ evaluations were performed with the percentage equality threshold derived from $K_{all}$ independent studies (5%).

Users of $K_{all}$ and $K_{strong}$ are generally interested in the evaluation of inner consistency aspects of p-tools predictions. In this regard, both $K_{all}$ and $K_{strong}$ rely on the consistency of preferences exhibited by pairs of p-tools across pairs of mutations. However, consistent preferences might hide quite different mutation effects. Without loss of generality, let us assume a common output scale for the p-tools *S* and *P*, and let us consider the mutations $m_{i}$ and $m_{j}$, i<j≤n. In addition, let us assume pairs of predictions $t_{S}\left(m_{i}\right)=0.11$ and $t_{S}\left(m_{j}\right)=0.12$ issued by *S*, and $t_{P}\left(m_{i}\right)=0.91$ and $t_{S}\left(m_{j}\right)=0.92$ issued by *P*, so that $r_{S}\left(m_{i},m_{j}\right)=r_{P}\left(m_{i},m_{j}\right)=1$ holds. Although both *S* and *P* predict that $m_{i}$ is less damaging than $m_{j}$, the pairs of predictions are in opposite ranges of the scale and involve quite different effects: While $m_{i}$ and $m_{j}$ might be benign according to *S*, they are both pathogenic according to *P*. This toy example points out that inner consistency measurements between pairs of p-tools may require the evaluation of multiple aspects, from the consistency of pairwise preferences to the consistency of the semantics behind individual predictions.

Aiming to shed light on the semantic aspect of p-tools inner consistency measurements, the Spearman’s rank correlation coefficient was considered. Briefly, the Spearman’s correlation [24] between two variables equals the Pearson’s correlation between the rank values of the two variables. However, while the Pearson’s correlation assesses only linear relationships, the Spearman’s correlation assesses general monotonic relationships, whether linear or not. For *n* distinct mutations, Spearman’s rank ($\rho_{s}$) correlation coefficient is associated to predictions issued by p-tools *S* and *P* can be computed using the following popular formula:

(4) $$\begin{array}{c} \rho_{s}=1-\frac{6*\sum d_{i}^{2}}{n*(n^{2}-1)} \end{array}$$

where $d_{i}$ is the difference between the ranks assigned to the *i-th* mutation by *S* and *P*, i≤n. In the case of identical predictions, the average value of their ascending ranking positions is used. Although correlation coefficients are intended to measure the “strength of pairwise relationships”, they might be confused by unclear rankings like those induced by p-tools with numerical outputs. On the other hand, although neither the $K_{all}$ nor the $K_{strong}$ indices consider the absolute position of p-tool predictions, i.e., their semantic aspect, they are not confused by small differences in numerical prediction values due to the introduction of the equality threshold for preference relationships. As a result, both $K_{all}$ and $K_{strong}$ are good candidates for making productive evaluations of p-tools inner consistency aspects.

### 2.4. Outer Consistency Analysis

Standard information retrieval metrics including the accuracy, the precision, the *recall*, the F1-score, and the Matthews correlation coefficient (MCC) were considered to evaluate the outer consistency of p-tools:

(5) $$accuracy=\frac{TP+TN}{TP+TN+FP+FN}$$

(6) $$precision=\frac{TP}{TP+FP}$$

(7) $$recall=\frac{TP}{TP+FN}$$

(8) $$F1-\mathrm{score}=\frac{2TP}{2TP+FP+FN}$$

(9) $$MCC=\frac{TP*TN-FN*FP}{\sqrt{\left(TP+FN)(TP+FP)(TN+FN)(TN+FP\right)}}$$

where TP, TN, FP, and FN stand for the number of true positive, true negative, false positive, and false negative predictions respectively. It is worth noting that special care should be taken with the above metrics when analyzing highly imbalanced datasets like those induced in experiments involving the high throughput screening of genetic mutations. Fortunately, the human being is a highly robust system, thus we expect most of the SNVs to be negative examples (benign mutations). Therefore, the accuracy is not a good metric for measuring the outer consistency of p-tools as a naive predictor set to predict only TN mutations would achieve a very high accuracy. On the other hand, the precision metric is useful to measure the proportion of mutations predicted as positive examples that were indeed TP predictions (pathogenic mutations).

Similarly, the recall metric is useful to measure the proportion of positive examples that were indeed TP predictions, with respect to the ground truth for positive examples. Both the precision and recall metrics disregard TN predictions. There is also often an inverse relationship between the precision and recall metrics so that it is possible to increase one of them at the expense of reducing the other; the F1-score, originally defined for document classification problems where TN predictions also do not matter, is defined as the harmonic mean of the precision and recall metrics. Finally, the MCC is a statistic robust to differences in the proportion of negative and positive examples that can be more appropriate than the F1-score when negative examples matter is some way. The MCC is called a correlation coefficient because it is −1 when predictions are completely wrong, 1 when they are completely correct, and 0 when they are not better than random predictions.

In order to analyze the outer consistency of p-tools, their outputs were binarized. Align GVGD predictions in “C0” and “C15” classes were considered negative examples (benign) and predictions in the “C45”, “C55”, “C65” classes were considered positive ones (pathogenic). Similarly, Provean predictions in the “Neutral” class were considered negative examples and predictions in the “Deleterious” class were considered positive ones. On the other hand, Panther predictions in the “Benign” class were considered negative examples and predictions in the “Damaging” class were considered positive ones. For Strum and Cupsat, predictions with ΔΔG >=0 were considered negatives examples, while predictions with ΔΔG <0 were considered positive ones. Finally, Polyphen2 predictions in the “Benign” class were considered negative examples and predictions in the “Probably” class were considered positive ones. In all the cases, p-tool predictions involving intermediate categories were disregarded for the outer consistency analysis.

## 3. Results and Discussion

### 3.1. Inner Consistency Results

Inner consistency measurements accomplished by means of the $K_{all}$ and $K_{strong}$ indices are shown in Table 1 and Table 2, respectively. The most “similar” and the most “different” p-tools identified by the $K_{all}$ and the $K_{strong}$ indices respectively, are highlighted in bold. Based on $K_{all}$, the Provean and Align GVGD are the most similar p-tools. Based on $K_{strong}$, the Polyphen2 and Align GVGD are the most different p-tools. As expected, $K_{strong}$ achieve larger values than $K_{all}$; this is reasonable as $K_{strong}$ only considers opposite preference relationships. The P-tools abbreviations are: Provean (Prov), Align GVGD (Gvgd), Cupsatd (Cupd), Cupsatt (Cupt), Panther (Pthr), and Strum (Strm).

In addition, Table 3 shows the inner consistency measurements accomplished by the Spearman’s correlation coefficient. These results show that many of the p-tools are poorly correlated. In principle, this may be attributed to differences in the semantic of predictions in each p-tool scale and/or the sensitivity of the Spearman’s correlation coefficient to p-tools with numerical outputs. For both the *BRCA1-SGE* and *BRCA1-HDR* datasets, the most correlated p-tools are Provean and Align GVGD, whose correlation coefficients are highlighted in bold. This is reasonable as both p-tools use sequence alignments to predict the effect of mutations.

**Figure 2:**
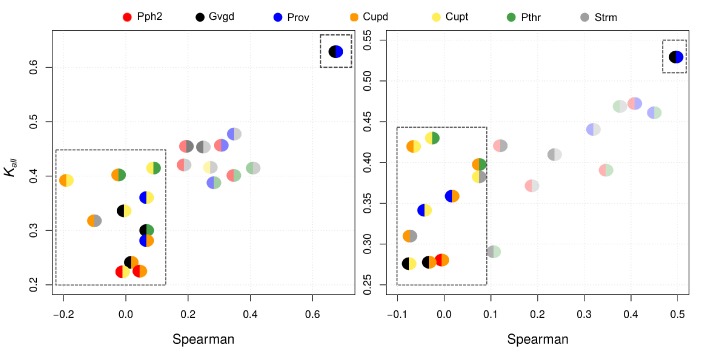
$K_{all}$ vs. Spearman for the *BRCA1-SGE* (**left**) and *BRCA1-HDR* (**right**) datasets. The top-right rectangles point out the most correlated p-toleftols. The bottom-left rectangles point out the Cupsatt/d correlations.

**Figure 3:**
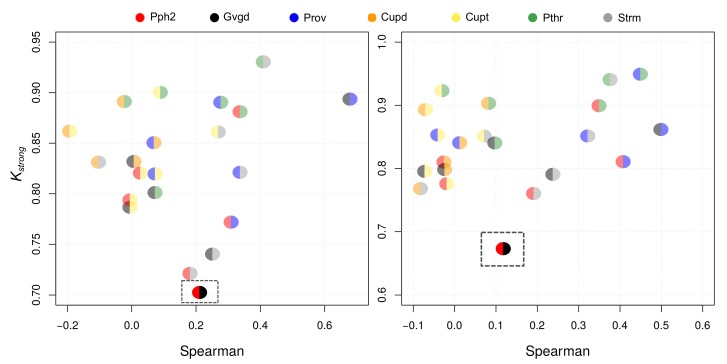
$K_{strong}$ vs. Spearman for the *BRCA1-SGE* (**left**) and *BRCA1-HDR* (**right**) datasets. The bottom rectangles point out the less correlated p-tools according to $K_{strong}$.

To shed light on the type of inner consistency information that $K_{all}$ and $K_{strong}$ are able to provide, we analyzed them against the Spearman’s correlation coefficient. In Figure 2, $K_{all}$ and Spearman appear related to each other in some degree. We note, however, that while both Cupsatt and Cupsatd are poorly correlated with almost all the other p-tools according to Spearman, they are close to many other p-tools according to $K_{all}$. On the other hand, both $K_{all}$ and Spearman show that Provean and Align GVGD are highly correlated. Finally, Figure 3 shows that $K_{strong}$ and Spearman are clearly uncorrelated. Remarkably, while $K_{strong}$ identifies Polyphen2 and Align GVGD as the most different p-tools, Spearman identifies Cupsatd and Cupsatt as the most negatively correlated ones. Although the $K_{strong}$ result makes sense since Polyphen2 and Align GVGD use different learning strategies and information sources, the Spearman result does not make sense since Cupsatd and Cupsatt are variations of the same algorithm (Cupsat) on the same information source.

### 3.2. Outer Consistency

The measurement of p-tools outer consistency is shown in Table 4. Only the information about TP and TN predictions is shown together with the MCC and F1-score statistics. Accuracy, precision, and recall metrics are shown in Appendix A.

The *BRCA1-SGE* dataset has a rather imbalanced distribution of positive and negative samples (G=−0.56). Three of the p-tools, Align GVGD, Cupsatt, and Panther, correctly predict more than 78% of the positive examples (TP). However, only Panther reasonably predicts negative ones (57%). We note, however, that the three p-tools also introduce many false positive predictions (see Appendix B). Based on the MCC and the F1-score, we can say that the best compromise in the prediction performance is achieved by Panther. The *BRCA1-HDR* dataset is highly imbalanced (G=−0.88). For this dataset, four of the p-tools, Align GVGD, Cupsatt, Panther, and Strum, correctly predict most of the positive examples (TP). However, only Panther reasonably predicts negative ones (74%). Based on the MCC and the F1-score, none of the p-tools achieved an acceptable prediction performance. This may be due to many false positive predictions (300 on average) with only 59 TP (see Appendix A). Provean does not predict any mutations as positive, making the F1-score and MCC equal to 0. On the whole, Panther achieves the best compromise in prediction performance for the considered p-tools on average. However, its prediction performance remains poor. Finally, our results show that although most of the mutations reported for the *BRCA1* gene are of the benign type, p-tools tend to classify them as pathogenic from the observed high rates of false positive predictions.

## 4. Conclusions

A number of bioinformatics tools have been developed to predict the impact of SNVs on the functionality of protein coding genes. The stronger the agreement between tools that use different prediction approaches and independent sources of information, the greater the confidence we can have in their predictions. Evaluating the level of confidence is particularly important when predictions are used to guide experimental research studies or clinical decisions. In this paper, a computational framework for evaluating the confidence of six tools that predict the impact of SNVs on protein coding genes has been presented. With this aim, two indices called $K_{all}$ and $K_{strong}$ have been introduced. The proposed indices can evaluate the consistency of predictions issued by different tools (inner consistency) without requiring the specific understanding of their outputs. Using these indices, the most similar and most different prediction tools can be identified. As a result, these indices can help to accelerate the understanding of new prediction tools. Last, these indices can help to design truly diverse ensembles of prediction tools, a fundamental requirement for improving the confidence of individual members of the ensemble.

Inner consistency studies were complemented with outer consistency studies focusing on the extent to which predictions matched the experimental results reported in literature. Without loss of generality, experimental data involving the high throughput screening of genetic mutations on the *BRCA1* gene were considered. The outer consistency studies confirmed the importance of selecting suitable information retrieval metrics since reference datasets are expected to be highly imbalanced. In general, the prediction performance of the tools was rather low with a clear trend towards the introduction of false positive predictions. On the whole, our results highlight the importance of understanding the intrinsic limitations of tools dealing with the prediction of SNV effects on protein coding genes.

## Figures and Tables

**Table 1:** The inner consistency between pairs of p-tools measured by the $K_{all}$ index, set to work with a 5% percentage equality threshold. The elements above the diagonal correspond to the *BRCA1-SGE* dataset while the elements below it correspond to the *BRCA1-HDR* dataset. The P-tools abbreviations are: Polyphen(Pph2), Provean (Prov), Align GVGD (Gvgd), Cupsatd (Cupd), Cupsatt (Cupt), Panther (Pthr), and Strum (Strm).

	Pph2	Prov	Gvgd	Cupd	Cupt	Pthr	Strm
Pph2		0.46	0.45	0.23	0.33	0.40	0.42
Prov	0.47		**0.62**	0.29	0.36	0.39	0.47
Gvgd	0.42	**0.52**		0.24	0.33	0.30	0.45
Cupd	0.28	0.36	0.28		0.39	0.40	0.32
Cupt	0.28	0.34	0.28	0.42		0.41	0.41
Pthr	0.37	0.46	0.29	0.39	0.43		0.41
Strm	0.39	0.44	0.41	0.31	0.38	0.47	

**Table 2:** The inner consistency between pairs of p-tools measured by the $K_{strong}$ index set to work with a 5% percentage equality threshold. The elements above the diagonal correspond to the *BRCA1-SGE* dataset while the elements below it correspond to the *BRCA1-HDR* dataset.

	Pph2	Prov	Gvgd	Cupd	Cupt	Pthr	Strm
Pph2		0.77	**0.70**	0.82	0.79	0.88	0.72
Prov	0.81		0.89	0.85	0.82	0.89	0.82
Gvgd	**0.67**	0.85		0.83	0.79	0.80	0.74
Cupd	0.81	0.84	0.80		0.86	0.89	0.83
Ccupt	0.78	0.85	0.79	0.89		0.90	0.86
Pthr	0.90	0.95	0.84	0.90	0.92		0.93
Strm	0.76	0.85	0.79	0.77	0.85	0.94	

**Table 3:** The inner consistency results for Spearman’s correlation coefficient. The elements above the diagonal correspond to the *BRCA1-SGE* dataset while the elements below it correspond to the *BRCA1-HDR* dataset.

	Pph2	Prov	Gvgd	Cupd	Cupt	Pthr	Strm
Pph2		0.31	0.20	0.03	0.00	0.34	0.18
Prov	0.41		**0.68**	0.07	0.07	0.28	0.34
Gvgd	0.12	**0.50**		0.01	0.00	0.07	0.25
Cupd	−0.03	0.01	−0.03		−0.19	−0.02	−0.10
Cupt	−0.02	−0.04	−0.07	−0.07		0.09	0.27
Pthr	0.35	0.45	0.09	0.08	−0.03		0.41
Strm	0.19	0.32	0.24	−0.08	0.07	0.38	

**Table 4:** The outer consistency of p-tools.

	*BRCA1-SGE*				*BRCA1-HDR*			
Tools	TP (#387)	TN (#1405)	MCC	F1-Score	TP (#59)	TN (#977)	MCC	F1-Score
Pph2	115	871	0.25	0.41	12	555	0.17	0.20
Prov	3	1404	0.06	0.02	0	977	0.00	0.00
Gvgd	343	198	0.16	0.42	55	21	0.01	0.11
Cupd	111	1031	0.07	0.28	25	195	0.19	0.37
Cupt	305	393	0.08	0.38	50	71	0.12	0.35
Pthr	319	803	0.33	0.49	51	723	0.31	0.28
Strm	114	111	0.19	0.41	56	244	0.11	0.13

## Appendix A. P-Tools Main Features

**PolyPhen-2**http://genetics.bwh.harvard.edu/pph2/-(Polymorphism Phenotyping v2) is a software tool for predicting the possible impact of an amino acid substitution on the structure and function of a human protein. It is based on a number of sequence, phylogenetic, and structural features characterizing the substitution. Predictions are performed by a naïve Bayesian classifier. The sequence-based features include position-specific independent Count (PSIC) scores, multiple sequence alignment (MSA) properties, and the position of mutations with respect to domain boundaries as defined by Pfam [25]. The structure-based features include solvent accessibility, changes in solvent accessibility for buried residues, and crystallographic B-factor. Two pair of datasets, namely HumDiv and HumVar, can be used for the generation of the corresponding classification models. The default classification model uses HumDiv data and is preferred for evaluating rare alleles, dense mapping of regions identified by genome-wide association studies, and analysis of natural selection. The HumVar classification model is better suited for the diagnostics of Mendelian diseases which require distinguishing mutations with drastic effects from all the remaining human variations, including abundant mildly deleterious alleles.

The PolyPhen-2 output is a table with a classifier label of the type benign/possible damaging/ probably damaging, a classifier probability of the mutations being damaging, a classifier model False Positive Rate (1-specificity) at the above probability, and a classifier model True Positive Rate (sensitivity) at the above probability. In this work, the probabilities of the mutations being damaging were considered for the inner consistency analysis and a binarization of categorical outputs was used for the outer consistency analysis. In both studies, the HumVar classification model was selected.

**Provean**http://sift.jcvi.org/-(Protein Variation Effect Analyzer v1.1.3) is a software tool for predicting whether an amino acid substitution has an impact on the biological function of a human or mouse protein. It is based on the change, caused by a given variation, in the similarity of the query sequence to a set of its related protein sequences. For this prediction, the algorithm is required to compute a semi-global pairwise sequence alignment score between the query sequence and each of the related sequences. This alignment-based score measures the change in sequence similarity of a query sequence to a protein sequence homolog before and after the introduction of an amino acid variation to the query sequence. The output prediction information of this tool is a table with a prediction label column and a score column. If the score is equal to or below a predefined threshold (e.g., −2.5), the protein variant is predicted to have a “deleterious” effect. If the score is above the threshold, the variant is predicted to have a “neutral” effect.

**Align GVGD**http://agvgd.hci.utah.edu/-(Grantham Variation and Grantham Deviation) is a software tool that combines the biophysical characteristics of amino acids and protein multiple sequence alignments to predict where missense substitutions in genes of interest fall in a spectrum from enriched deleterious to enriched neutral. The output prediction information of this tool is a table with a score column that represents an extension of the Grantham difference, to score missense substitutions against the range of variations present at their position in a multiple sequence alignment and a categorical column with seven classes ordered from most likely to interfere with function to least likely.

**Strum**https://zhanglab.ccmb.med.umich.edu/STRUM/-(Structure based Prediction of Protein Stability Changes Upon Single-point Mutation) is a software tool for predicting the fold stability change (ΔΔG) of protein molecules upon single-point mutations. Strum adopts a gradient boosting regression approach to train the Gibbs free-energy changes on a variety of features at different levels of sequence and structure properties. The unique characteristic of Strum is the combination of sequence profiles with low-resolution structure models from protein structure prediction, which helps to enhance the robustness and accuracy of the method and make it applicable to various protein sequences, including those without experimental structures. The output prediction information of this tool is a column with the ΔΔG value of each mutation.

**Cupsat**http://cupsat.tu-bs.de-(Cologne University Protein Stability Analysis Tool) is a software tool for predicting changes in protein stability upon point mutations. It uses structural environment specific atom potentials and torsion angle potentials to predict ΔΔG, the difference in free energy of unfolding between wild-type and mutant proteins. To improve accuracy and specificity of predictions, the mutations and mean-force potentials were classified according to different structural regions. Initially, the secondary structure specificity of mutations and mean-force potentials was implemented and the amino acids were classified into helices, sheets, and others. Later, the amino acids belonging to each of these secondary structure elements were further subdivided according to their solvent accessibility.

This method requires the primary and secondary structure information (PDB file) and can be run with two different experimental methods: thermal and denaturants, referred as **Cupsatt** and **Cupsatd** within the manuscript, respectively. The output prediction information is a table with a categorical column indicating the overall stability of the mutation (Stabilising or Destabilising), a categorical column with the torsion information of the mutation (Favourable or Unfavourable) and a numerical column of the Predicted ΔΔG (kcal/mol) value. The numerical information was used in our analysis. Some amino acids of the BRCA1 structure were not present in the PDB file used (ID: 1jm7 and 4y2g) and therefore were not considered in the comparisons.

**Panther**http://www.pantherdb.org/-(Protein Analysis Through Evolutionary Relationships v15.0) is a software tool that calculates substitution position-specific evolutionary conservation (subPSEC) scores based on alignments of evolutionary related proteins to predict the pathogenicity. The alignments are obtained from the PANTHER library of protein families based on Hidden Markov Models (HMMs). The subPSEC score describes the amino acid probabilities, and in particular, positions among evolutionary related sequences. The output prediction information of this tools is a categorical column indicating whether the mutation may or may not affect the functionality of the protein.

## Appendix B. Outer Consistency

**Table A1:** The outer consistency results for the *BRCA1-SGE* dataset.

Tools	Acc.	Prec.	Recall	MCC	F1-Score
Pph2	0.75	0.38	0.44	0.25	0.41
Prov	0.79	0.75	0.01	0.06	0.02
Gvgd	0.36	0.27	0.96	0.16	0.42
Cupd	0.67	0.28	0.29	0.07	0.28
Cupt	0.41	0.25	0.29	0.08	0.38
Pthr	0.63	0.35	0.82	0.33	0.49
Strm	0.40	0.26	0.93	0.19	0.41

**Table A2:** The outer consistency results for the *BRCA1-HDR* dataset.

Tools	Acc.	Prec.	Recall	MCC	F1-Score
Pph2	0.86	0.14	0.39	0.17	0.20
Prov	0.94	0.00	0.00	0.00	0.00
Gvgd	0.08	0.06	0.98	0.01	0.11
Cupd	0.72	0.32	0.37	0.19	0.37
Cupt	0.40	0.22	0.85	0.12	0.35
Pthr	0.75	0.17	0.85	0.31	0.28
Strm	0.07	0.07	0.95	0.11	0.13
